# Extraction of a Large Stone by the Lateral Operation

**Published:** 1847-02

**Authors:** R. Haywood

**Affiliations:** Tuscaloosa, Alabama


*Extraction of a large stone by the lateral operation.* By R. Haywood, M. D. of Tuscaloosa, Alabama, (communicated in a letter to Professor Pancoast.)

Dear Sir:—I take pleasure in reporting to you another operation of Lithotomy which I have performed successfully. * * *

I was consulted by Mr. Armstead, of Tuscaloosa county, thirteen miles from this place, in a case of stone in his son, that had manifested unequivocal signs of its existence from birth. I sounded him and found a calculus of large size. I prepared him for an operation as soon as the condition of his case would permit, his health having suffered greatly from the presence of this foreign body in the bladder, until he was in his thirteenth year. As soon as the necessary preparation was made I performed the lateral operation, and extracted a stone weighing 1 oz. 5 dr. 16 grs.

I should have completed the whole operation in six minutes, had it not been that the shape of the stone was oblong, and I seized it first in its longitudinal diameter. I changed the forceps, and then seized it by its transverse diameter and extracted it.

In five weeks from the operation I sent the boy home, perfectly well. His health is now good, and he is daily improving in strength.

I send you enclosed an analysis of the stone and its weight, shape, size, appearance, &c., by Professor Brumby of the University of Alabama, (a gentleman highly distinguished in our state,) which you will be much gratified to examine.

*University of Alabama, September 23d,* 1846.

Dr. R. Haywood.

Dear Sir:—I have made a careful chemical examination of the calculus, which you gave me for the purpose on the 21st inst.

It was the largest I ever saw; weighed 653.70 grs., Troy, though three small fragments had been detached from it, while in your possession; was oblong-oval in form, slightly flattened; in colour yellowish-brown externally, white, with a tinge of yellow internally; its surface presented very distinct tubercles, and was coated with small, white, shining crystals. It was solid, compact, brittle, and easily cut or scraped with a knife; and when divided by a very fine saw, in the direction of the larger diagonal, its structure was beautifully displayed, showing a series of concentric coats, that diminished in hardness to the small, pulverulent, central nucleus.

Though only partially soluble in cold, it softened readily in hot water, and formed a white gelatinous mass, which subsided as the water cooled. In a solution, cold or hot, of pure potassa, it was insoluble; but it dissolved readily in dilute nitric, sulphuric, hydrochloric, and acetic acids. From these acid solutions, it was readily precipitated by excess of pure potassa or soda with the evolution of a urinous, ammoniacal odour. A few drops of ammoniacal nitrate of silver being added to a neutral solution of it in nitric acid, the peculiar colour of phosphate of silver instantly appeared. A fragment heated to redness in a small platinum crucible first turned black, then became white, and was neither consumed nor fused; and, even before the blowpipe, the white residue was fused without difficulty. I was unable, by the most careful experiments, to detect the presence of uric acid, in any sensible quantity.

You will perceive, from these results, that it must belong to the species denominated by chemists *ammoniaco-magnesian phosphate* or *triple calculus.* This species is rarely pure, but generally contains a mixture of phosphate of lime. I did not deem it necessary to investigate this point, as its determination would not have any influence on your treatment of your patient.

Yours most respectfully,

R. T. Brumby.

